# Different localization of P2X4 and P2X7 receptors in native mouse lung - lack of evidence for a direct P2X4-P2X7 receptor interaction

**DOI:** 10.3389/fimmu.2024.1425938

**Published:** 2024-06-17

**Authors:** Juan Sierra-Marquez, Lena Schaller, Lukas Sassenbach, Antonio Ramírez-Fernández, Philipp Alt, Björn Rissiek, Béla Zimmer, Johann Schredelseker, Julia Hector, Tobias Stähler, Friedrich Koch-Nolte, Claudia A. Staab-Weijnitz, Alexander Dietrich, Robin Kopp, Annette Nicke

**Affiliations:** ^1^ Walther Straub Institute of Pharmacology and Toxicology, Member of the German Center for Lung Research (DZL), Faculty of Medicine, LMU Munich, Munich, Germany; ^2^ Department of Neurology, University Medical Centre Hamburg-Eppendorf, Hamburg, Germany; ^3^ Deutsches Zentrum für Herz-Kreislauf-Forschung, Partner Site Munich Heart Alliance, Munich, Germany; ^4^ Institute of Immunology, University Medical Centre Hamburg-Eppendorf, Hamburg, Germany; ^5^ Institute of Lung Health and Immunity (LHI), Helmholtz Munich, Comprehensive Pneumology Center (CPC-M), Member of the German Center for Lung Research (DZL), Germany; ^6^ Department of Pediatrics, University of Colorado Anschutz Medical Campus, Aurora, CO, United States

**Keywords:** P2X7 receptor, P2X4 receptor, heteromerization, functional interaction, lung epithelial cells, macrophage, nanobody, BAC transgenic P2X7-EGFP mouse

## Abstract

**Introduction:**

P2X receptors are a family of homo- and heterotrimeric cation channels gated by extracellular ATP. The P2X4 and P2X7 subunits show overlapping expression patterns and have been involved in similar physiological processes, such as pain and inflammation as well as various immune cell functions. While formation of P2X2/P2X3 heterotrimers produces a distinct pharmacological phenotype and has been well established, functional identification of a P2X4/P2X7 heteromer has been difficult and evidence for and against a physical association has been found. Most of this evidence stems, however, from *in vitro* model systems.

**Methods:**

Here, we used a P2X7-EGFP BAC transgenic mouse model as well as P2X4 and P2X7 knock-out mice to re-investigate a P2X4-P2X7 interaction in mouse lung by biochemical and immunohistochemical experiments as well as quantitative expression analysis.

**Results:**

No detectable amounts of P2X4 could be co-purified from mouse lung via P2X7-EGFP. In agreement with these findings, immuno-histochemical analysis using a P2X7-specific nanobody revealed only limited overlap in the cellular and subcellular localizations of P2X4 and P2X7 in both the native lung tissue and primary cells. Comparison of P2X4 and P2X7 transcript and protein levels in the respective gene-deficient and wild type mice showed no mutual interrelation between their expression levels in whole lungs. However, a significantly reduced *P2rx7* expression was found in alveolar macrophages of *P2rx4* ^-/-^ mice.

**Discussion:**

In summary, our detailed analysis of the cellular and subcellular P2X4 and P2X7 localization and expression does not support a physiologically relevant direct association of P2X4 and P2X7 subunits or receptors *in vivo*.

## 1 Introduction

The P2X family of trimeric ion channel receptors comprises seven subtypes, P2X1-P2X7, of which all but the P2X6 subunit can form functional homomeric ion channels on their own (1). While a variety of possible P2X heteromers have been characterized *in vitro*, only few of them (such as P2X2/3 (2), P2X1/5 (3), and P2X2/5 (4)) have been confirmed *in vivo* also see (5–7). The P2X7 subtype differs structurally and functionally from other P2X receptors. The most significant differences are the presence of a palmitoylated cytoplasmic membrane anchor and a large intracellular so-called “ballast” domain (8), a low sensitivity to ATP (1), and its ability to initiate various downstream effects upon activation, such as the formation of large membrane pores (9).

Within the P2X receptor family, P2X4 and P2X7 are the most closely related subunits (47% and 48% amino acid sequence identity for the human and mouse proteins, respectively) and in humans and rodents both genes are direct neighbors on the same chromosome (10, 11). P2X4 and P2X7 are also co-expressed in many cell types including microglia (12), macrophages (13, 14), T cells (15), different cell types in the lung (16, 17) and secretory cells (18). While native P2X4 appears to be predominantly localized in lysosomes in many cell types (19), both subunits have been linked to similar processes, such as release of IL-1β and IL-18 and production of reactive oxygen species (ROS) (20–24), phagosome function (19, 25), autophagy, macrophage death (26), autocrine and paracrine activation of T cells (15, 27–31), and secretion of lung surfactant (32, 33).

Based on electrophysiological recordings, a functional P2X4/P2X7 interaction was originally suggested in airway ciliated cells (16) and subsequently described for the heterologously expressed subunits in HEK293 cells (14, 34). These studies are supported by measurements of dye uptake, where a positive effect of P2X4 on pore formation was identified in mouse macrophages (24, 26) while a negative P2X4 effect was observed in co-transfected HEK293 cells (34). However, neither a more recent study in *Xenopus laevis* oocytes, where both subunits were heterologously co-expressed (35), nor a detailed pharmacological analysis of endogenous subunits in BV-2 microglia did find evidence for a functional interaction (36).

A physical interaction of P2X4 and P2X7 subunits has been shown by co-purification experiments using transfected HEK293 and tsA201 cells as well as in mouse bone marrow-derived macrophages, the E10 mouse alveolar epithelial cell line, and primary gingival epithelial cells (14, 22, 24, 37–39). Further analysis by cross-linking, native polyacrylamide gel electrophoresis (PAGE) and atomic force microscopy indicated that the receptors formed complexes of interacting homotrimers rather than heterotrimers (37–39). In support of these studies, a close association of co-expressed P2X4 and P2X7 subunits was detected by Förster resonance energy transfer (FRET) analysis in *Xenopus laevis* oocytes and co-transfected HEK293 cells (24, 35) as well as in an *in situ* proximity ligation assay in HEK cells (39). Blue native PAGE analysis of P2X4 and P2X7-containing complexes across a variety of mouse tissues and a systematic co-precipitation study in HEK cells failed, however, to identify P2X4/P2X7 complexes that survived solubilization (5, 40).

A mutual interaction of both subunits was also reported at the transcriptional/translational level: in mouse kidney, a significant reduction of *P2rx4* or *P2rx7* mRNA levels was observed if the gene of the respective other P2X subtype was deleted (41). Likewise, P2X4 deficiency in bone marrow-derived dendritic cells led to reduced *P2rx7* mRNA levels and decreased IL-1β release upon ATP treatment (42) and co-transfection of P2X7 and P2X4 increased surface expression of P2X4 in normal rat kidney (NRK) cells while total P2X4 levels remained unchanged (14). In mouse E10 alveolar epithelial cells, in contrast, downregulation of one subtype via shRNA resulted in an increased protein level of the respective other subtype (38). In RAW264.7 macrophage-like cells and bone marrow-derived dendritic cells, however, shRNA-mediated downregulation of P2X4 did not affect P2X7 protein levels (23, 26) and most recently, evidence against an interdependent regulation or activation of both receptors and a heteromeric assembly was shown in the murine BV-2 microglia cell line (36).

Both subtypes are involved in immune cell function and are expressed in the lung where they have been shown to play a role in inflammatory processes (43, 44) and surfactant secretion (32, 33). Both have also been involved in a variety of pulmonary diseases, like asthma, acute lung injury (ALI), and chronic obstructive pulmonary disease (COPD) (42, 45–47). Due to its low ATP sensitivity, the proinflammatory P2X7 receptor is assumed to be mainly activated under pathophysiological conditions and it is therefore considered an interesting drug target (48, 49). In contrast, the P2X4 receptor has been shown to serve also important physiological functions such as blood pressure regulation and cardiac myocyte contractility (50, 51). Its blockade, while shown to be beneficial in pain states (52), is therefore expected to cause unwanted side effects.

Thus, considering their physiological and potential pathophysiological roles and their potential to serve as drug targets, it is important to better understand the physiological relevance of their interaction and in particular, the possibility of heteromer formation as this would enable the development of subtype-specific antagonists. Here, we set out to reinvestigate this interaction in native mouse lung using a P2X7-EGFP overexpressing reporter mouse as well as P2X4 and P2X7-deficient mice (*P2rx4^-/-^, P2rx7^-/-^*).

## 2 Materials and methods

### 2.1 Animals

Tg(RP24–114E20P2X7451P-StrepHis-EGFP)Ani (P2X7-EGFP), *P2rx7* ^tm1d(EUCOMM)Wtsi^ (*P2rx7* ^-/-^), and *P2rx4* ^tm1Rass^ (*P2rx4* ^-/-^) mice have been described (53, 54). All mice were bred in a C57Bl/6N background and housed in standard conditions (22°C, 12 h light–dark cycle, water/food *ad libitum*). All animal experiments were performed in accordance with the principles of the European Communities Council Directive (2010/63/EU). Procedures were reviewed and approved by the Government of Upper Bavaria (ROB, 55.2–1-54–2532-59–2016, 55.2–2532.Vet_02–20-147). All efforts were made to minimize suffering and number of animals.

### 2.2 Protein extraction from mouse tissue

Mice were euthanized by isoflurane exposure followed by cervical dislocation. The lung was dissected and milled in 600 µl of homogenization buffer (0.1 M sodium phosphate buffer, pH 8.0, 0.4 mM Pefabloc SC (Sigma) and Complete protease inhibitor (Roche Applied Science) using a Precellys 24 homogenizer (Peqlab) and 2.8 mm ceramic beads. Cell fragments, nuclei, and organelles were pelleted by centrifugation at 1000 x g and 4°C for 15 min. The supernatant, comprising membrane fragments and soluble proteins, was subsequently centrifuged at 21000 x g and 4°C for 60 min to pellet the crude membrane fraction. Membrane proteins were solubilized by resuspension in extraction buffer containing 1% NP-40 (Sigma), 0.5% n-dodecyl-β-D-maltoside (Calbiochem) or 1% digitonin (Sigma) and incubated for 15 min at 4°C. The protein extract was afterwards cleared from insoluble fragments by centrifugation (21000 x g, 4°C, 10 min) to obtain the supernatant with solubilized membrane proteins.

### 2.3 Protein expression in and extraction from *Xenopus laevis* oocytes

P2X4 and P2X7-EGFP were subcloned into the pNKS2 oocyte expression vector (55). Linearized (*XbaI*) plasmid DNA was purified with the Qiagen clean up kit and cRNA was synthesized using the mMESSAGE mMACHINE SP6 transcription kit. *Xenopus laevis oocytes* were kindly provided by Prof. Luis Pardo (Max Planck Institute for Experimental Medicine, Göttingen), injected with 25 ng cRNA, and kept at 16°C in ND96, supplemented with 500 µl/ml gentamycin. 2–3 days after injection, 6–12 oocytes were homogenized in extraction buffer (0.1 M phosphate buffer (pH 8.0), 0.4 mM Pefabloc SC, and 1% NP40 or 0.5% n-dodecyl-β-D-maltoside, 20 µl buffer/oocyte). After 15 min incubation on ice, the protein extract was cleared by two centrifugation steps (10 min, 15000 x g, 4°C).

### 2.4 Protein expression in and extraction from HEK cells

P2X4 and P2X7 were subcloned into the pcDNA3.1 mammalian expression vector. Cells were seeded on 6-well plates at a density of 2 x 10^5^ cells per well in serum-free medium. The next day, DNA was introduced in the cells using the Turbofect transfection reagent (Thermo, Germany) following the indications of the manufacturer. Cells were kept at 37°C. 2 days after transfection, cells were detached by flushing them directly with sodium phosphate buffer (pH 8.0). The cells were pelleted by centrifugation at 800 x g for 5 min at 4°C, and later homogenized in 150–500 μl extraction buffer containing 0.1 M phosphate buffer (pH 8.0) supplemented with Pefabloc SC and 0.5% n-dodecyl-β-D-maltoside. After 15 min incubation on ice, the membrane fraction was obtained by centrifugation for 10 min at 21000 x g and 4°C and collection of the supernatant.

### 2.5 Immunoprecipitation

10–30 µl GFP-Trap^®^ agarose beads (Chromotek) were washed three times (1000 x g, 1 minute, 4°C) with washing buffer (1:5 dilution of extraction buffer in sodium phosphate buffer (pH 8.0) supplemented with 150 mM NaCl). 300 µl of protein extracts were added to the beads and incubated under slow rotation for 1 h at 4°C. Beads were then washed three times with 500 μl of washing buffer and purified protein eluted by 2 min incubation with 45 µl 0.2 M glycine (pH 2.5) and subsequent neutralization with 5 µl 1 M Tris (pH 10.5), as recommended by the manufacturer.

### 2.6 SDS-PAGE and western blot analysis

Proteins (40 μl extract, 20–40 μl eluate) were separated on 8% SDS-PAGE gels and blotted onto Immobilon-FL PVDF membranes (Merck Millipore) for 16 h at 4°C using a Mini Trans-Blot cell (Bio-Rad). After transfer, membranes were blocked with Intercept (TBS) Blocking Buffer (LI-COR Biosciences) diluted 1:2 in TBS. For the immunological detection of proteins, the membrane was incubated with the specific primary antibodies diluted in blocking buffer for 60 min at RT or overnight at 4°C. After washing three times for 5 min with TBS-T (0.1% Tween-20), the membrane was incubated with the fluorescent dye-conjugated secondary antibodies diluted in TBS-T for 60 min at RT. The membrane was again washed three times for 5 min with TBS-T and finally rinsed with TBS before detecting signals by using the Odyssey infrared imaging system (LI-COR Biosciences). For antibodies see **Supplementary Table S1**.

### 2.7 Nanobody production

7E2-rbIgG and 7E2-hIgG1 heavy chain antibodies (hcAbs) were generated by cloning the mouse P2X7-specific nanobody 7E2 upstream of the hinge and Fc of rabbit IgG or the hinge and Fc of human IgG1 into the pCSE2.5 vector respectively (vector was kindly provided by Thomas Schirrmann, Braunschweig, Germany (56). HcAbs were produced by transiently transfected HEK-6E cells cultivated in serum-free medium. Six days post transfection supernatants were harvested and hcAbs were purified by protein A Sepharose affinity chromatography as described earlier (57).

### 2.8 Immunofluorescence staining of lung frozen sections

Mice were euthanized by careful cervical dislocation and subsequently transcardially perfused with 20 ml PBS (pH 7.4) followed by 20 ml 4% PFA/PBS. The lungs were then intratracheally inflated with 1ml of 4% PFA/Tissue-Tek O.C.T. and the trachea subsequentially sealed with a suture. Lungs were removed, post-fixed overnight in 4% PFA/PBS (at RT), and cryoprotected for 24 h at 4°C in a 10–25% sucrose gradient in PBS (pH 7.4) before they were embedded in Tissue-Tek O.C.T and frozen at -20°C. 10 or 20 µm sections were prepared and dried for 30 min at RT on glass slides, followed by an antigen retrieval step (25 min incubation at 37–50°C in citrate buffer (10 mM sodium citrate, 0.05% Tween20, pH 6.0). Slices were then blocked for 1 h at RT (0.4% Triton X-100, 1% BSA, 5% normal goat serum (NGS) in PBS). Lung sections were afterwards incubated for 16–24 h at 4°C with primary antibodies in a humidified chamber, washed 5 x 10 min with PBS-T (0.05% Tween20 in PBS), and stained for 2 h at RT with fluorescent dye-conjugated secondary antibodies. All antibodies were diluted (for antibody details and ratios see **Supplementary Table S1**) in blocking solution. After washing (5 x 10 min, PBS-T), slices were incubated for 1–3 min with 4’, 6-diamidino-2-phenylindole (DAPI, 0.1 mg/l in PBS) and washed again (2 x 10 min, PBS). In some cases, Thiazole Red (TO-PRO-3, 1:1000, ThermoFisher Scientific) was used instead of DAPI and incubated together with the secondary antibodies. Coverslips were mounted using PermaFluor mounting medium, and slides were kept overnight at RT and then saved at 4°C until confocal scanning. For long-term storage, slices were kept at -20°C.

### 2.9 Preparation of alveolar macrophages

Mice were euthanized by cervical dislocation and lungs were then intra-tracheally lavaged (6x) with 1 ml PBS/0.5% BSA/2 mM EDTA (pH 7.4). Cells were collected by centrifugation (300 g at RT for 5 min), resuspended in RPMI1640 media containing 10% FCS, Pen/Strep (10 U/mL; 10 µg/ml) and 50 µM β-mercaptoethanol, and seeded in cell culture dishes (24-well for staining, and 5 cm for RNA preparation). After 12–24 h (37°C, 5% CO_2_) cells were washed in PBS and either fixed for immunofluorescence staining or lysed in Trizol for subsequent RNA extraction.

### 2.10 Isolation and culture of alveolar epithelial type 2 cells

Isolation of alveolar epithelial cells was performed based on former protocols (58, 59) with some minor modifications (60). In brief, 4–5 mice were euthanized by cervical dislocation and lungs were perfused with 0.9% NaCl (B. Braun Melsungen AG) and instilled with 1.5 ml dispase solution (Corning) followed by 400 μl 1% low-melting agarose (Sigma) via the trachea. Lungs were removed and digested in dispase for 45 min. Tissues were treated with DNAse I (AppliChem) in DMEM (ThermoFisherScientific) with HEPES (AppliChem) and were processed through 100 µm, 20 µm and 10 µm meshes (Sefar) to obtain a single cell suspension. After centrifugation, the cell pellet was resuspended in medium without DNAse I, transferred to cell culture dishes coated with antibodies directed against CD16, CD32 and CD45 (see **Supplementary Table S1**) and incubated for 30 min at 37°C to remove immune cells. The supernatant and medium from an additional wash step were collected, transferred to cell culture dishes and incubated for 60 min at 37°C to remove fibroblasts. The suspension was centrifuged at 200 g for 5 min, cells were resuspended in DMEM medium buffered with HEPES containing 10% FCS, and seeded in a six-well plate. AT2 cells were collected 36–48 h after seeding for IF-staining. Cell identity was confirmed with an antibody against the cell type-specific marker protein pro-SP-C (**Supplementary Table S1**).

### 2.11 Immunofluorescence staining of adherent cells

Cells plated on 1.3 cm glass coverslips were fixed with 4% PFA/PBS (10 min at RT), washed twice with PBS, permeabilized for 10 min or 1 h at RT with 0.5% Triton X-100/PBS, and blocked (4% BSA, 4% normal goat serum diluted in PBS) for 60 min at RT. Cells were then incubated with primary antibodies (16–24 h at 4°C, see **Supplementary Table S1**), fluorescence conjugated secondary antibodies (60 min at RT) and DAPI staining solution (0.2 mg/ml DAPI/PBS, 1–3 min at RT). After each labeling step, cells were washed in 0.1% BSA/PBS (5 x 5 min at RT) and finally rinsed in milli-Q H_2_O before they were mounted on object slides with PermaFluor mounting medium. All antibodies were diluted in 2% BSA/PBS (see **Supplementary Table S1**). Images were obtained by confocal laser scanning microscopy using a Zeiss LSM 880.

### 2.12 Confocal imaging

Confocal images were taken using a Zeiss LSM 880 microscope with airyscan using the ZEN Black software (2.3 SP1 FP3). Each channel was imaged separately to avoid bleed-through between the channels. Tissue/cell samples and respective negative controls from knock-out mice were prepared in parallel and used to determine background staining. The same settings (laser intensity and digital gain) were then applied for samples from wt and transgenic mice. Airyscan deconvolution was performed with automatic settings for all channels. Intensity profiles were created with the image processing suite from Zeiss ZEN 2.3 SP1 FP3 (black) at a representative position close to the nucleus. Images were processed with FIJI [Image J v.1.53c, Wayne Rasband, National Institutes of Health, USA, (61)].

### 2.13 RNA extraction and real-time PCR

Total mRNA was isolated from 20–30 mg of mouse tissue using the RNeasy plus mini kit (Qiagen). In the case of alveolar macrophages Trizol (ThermoFisher Scientific) was used for RNA extraction (1x10^6^ cells/ml). Complementary DNA (cDNA) was synthesized with the QuantiNova reverse transcription kit (Qiagen) following the manufacturer’s instructions from 1 μg of RNA. The quantitative PCR was carried out in a LightCycler 480 System using LightCycler 480 SYBR green I Master (Roche) and LightCycler 480 multiwell plates using 1 μl of cDNA. Primers were designed to span an exon-exon junction (see **Supplementary Table S2**). Relative mRNA levels were calculated by the ΔCt-method using ribosomal protein lateral stalk subunit P0 and peptidylprolyl isomerase A (PPIA) (whole lung) or only PPIA (alveolar macrophages) as a reference (62): Relative mRNA level = 2^-ΔCt^, where ΔCt = Ct(target)- Ct(reference) and Ct = cycle threshold.

### 2.14 Statistical analysis

Statistical analysis was performed with Graph Pad Prism software and data are presented as means ± standard deviation (SD). Shapiro-Wilk normality test was applied before mean comparison. After meeting the assumptions of normality and variance homogeneity, student’s t-test was used to determine statistical differences between groups. Significance was accepted at * p < 0.05, ** p < 0.01, *** p < 0.001 and **** p < 0.0001.

## 3 Results

### 3.1 P2X4 is not co-purified with transgenic P2X7-EGFP from mouse lung

**Figure 1:**
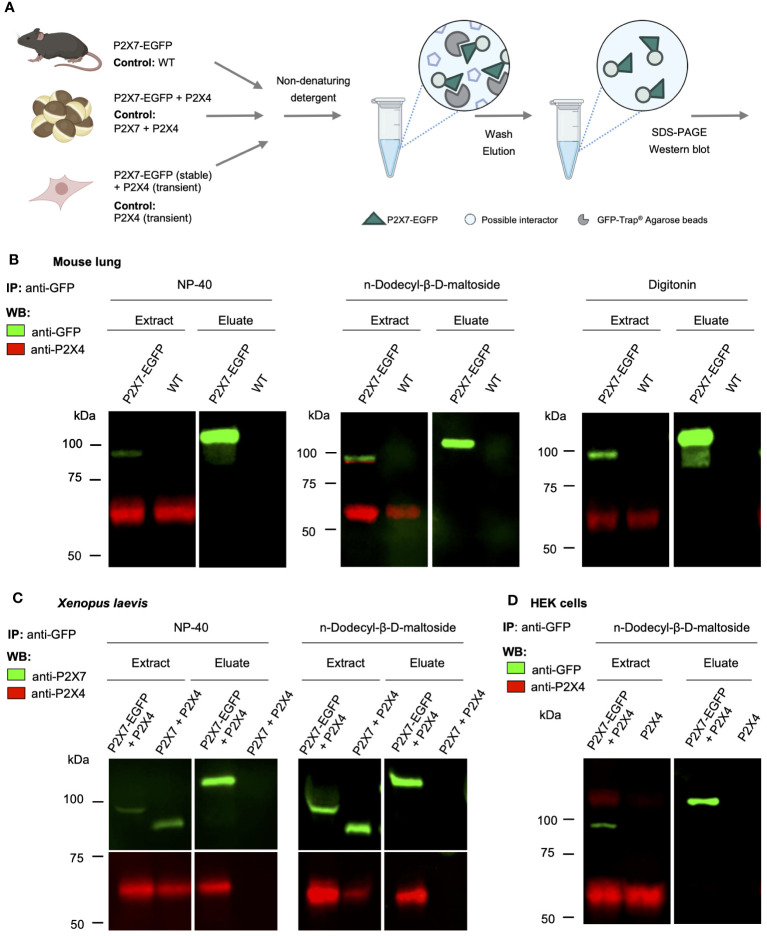
Lack of evidence for a physical interaction of P2X4 and P2X7 receptors in transgenic P2X7-EGFP mice. **(A)** Schematic representation of the purification of P2X7-containing complexes from mouse lung, cRNA-injected *Xenopus laevis* oocytes, and transfected HEK cells using anti-EGFP nanobodies (GFP-Trap^®)^. **(B)** Immunoprecipitation of P2X7-EGFP complexes from lung tissue of BAC-transgenic P2X7-EGFP mice and wt controls using the indicated non-denaturing detergents for solubilization. Primary antibodies against GFP (from rat) and P2X4 (from rabbit) and secondary antibodies for infrared imaging were used for blot development and detection. Note that P2X4 has a tendency to form higher aggregates (dimers band in the middle image) but does not associate with P2X7 (absence of a band stained with both antibodies). **(C)** Co-purification of P2X4 with P2X7-EGFP from *Xenopus laevis* oocytes. cRNA encoding P2X4 subunits was injected together with cRNA encoding P2X7-EGFP or non-tagged P2X7 subunits (negative control). After 2 days, P2X7-EGFP complexes were immunoprecipitated as in **B**, using the indicated detergents. Blots were developed with antibodies against P2X4 and P2X7 (both derived from rabbit). **(D)** Immunoprecipitation of P2X7-EGFP complexes from HEK293 cells. P2X4-encoding DNA was transfected transiently into HEK293 cells stably expressing P2X7-EGFP and wt control cells. Proteins were extracted in n-dodecyl-β-D-maltoside and purified and detected as in **(B)**. Representative results of at least two experiments are shown. Note that the harsh conditions required to elute the protein from the nanobody-coupled beads leads to a stronger protein denaturation than addition of SDS and results in a size shift of the P2X7-EGFP band as well as loss of EGFP fluorescence. Also note, that the eluate was about three times more concentrated than the extract. Panel **(A)** was created with BioRender.com.

*P2rx4* and *P2rx7* have been shown to be widely expressed in lung tissue (17) and the physical interaction of the respective proteins as well as a mutual interrelation has been reported in a murine alveolar epithelial cell line (38) and in mouse primary macrophages (24). To verify and quantify this interaction in native lung tissue, we used a BAC transgenic P2X7-EGFP reporter mouse model and performed pull-down experiments using the transgenic EGFP-tagged P2X7 receptor as a bait and bead-coupled nanobodies against GFP for purification (**Figure 1A**). As seen in **Figure 1B**, both P2X4 and P2X7 were clearly detected in extracts of the mouse lung, confirming their expression in this tissue. In contrast to the studies described above, however, no P2X4 subunits could be co-purified from lung extracts prepared with the non-denaturing detergents NP40, n-dodecyl-β-D-maltoside, and digitonin (**Figure 1B**). Since P2X4 was previously shown to associate closely with P2X7 in *Xenopus laevis* oocytes and to be purified with P2X7 from the tsA201 subclone of HEK cells (35, 39), we decided to use these same expression systems as a positive control and next co-expressed mouse P2X4 together with an EGFP-tagged mouse P2X7 construct in these heterologous expression systems. Using the same conditions as applied for the mouse tissue and an identical P2X7-EGFP construct, P2X4 could be clearly co-purified with P2X7 from *Xenopus* oocytes (**Figure 1C**) but not from HEK cells stably expressing the P2X7-EGFP construct and transiently co-transfected with P2X4 (**Figure 1D**). Based on these results, we argue that there is no significant physical interaction (either heteromerization of subunits or dimerization of receptors) between P2X4 and P2X7 in native mouse lungs.

### 3.2 Cell type-specific localization of P2X7 in the mouse lung

Presence of both P2X4 and P2X7 protein has been shown in different cell types in the lung (38, 63). These studies mostly relied on pharmacological analysis and transcript identification in cultured primary cells or cell lines. More recent single-cell RNA-sequencing (scRNA-Seq) studies (64) indicate that, except for macrophages, single cell expression data of the corresponding genes *P2rx4* and *P2rx7* display limited overlap in intensity and cell type distribution, suggesting a subordinate role of co-expression in mouse lung tissue (**Supplementary Figure 1A**). Transcript data, however, often do not correlate with protein abundances (compare **Supplementary Figure 1B**) and some cell types, e.g. alveolar type 1 (AT1) cells, are frequently underrepresented in scRNA-Seq data (65).

**Figure 2:**
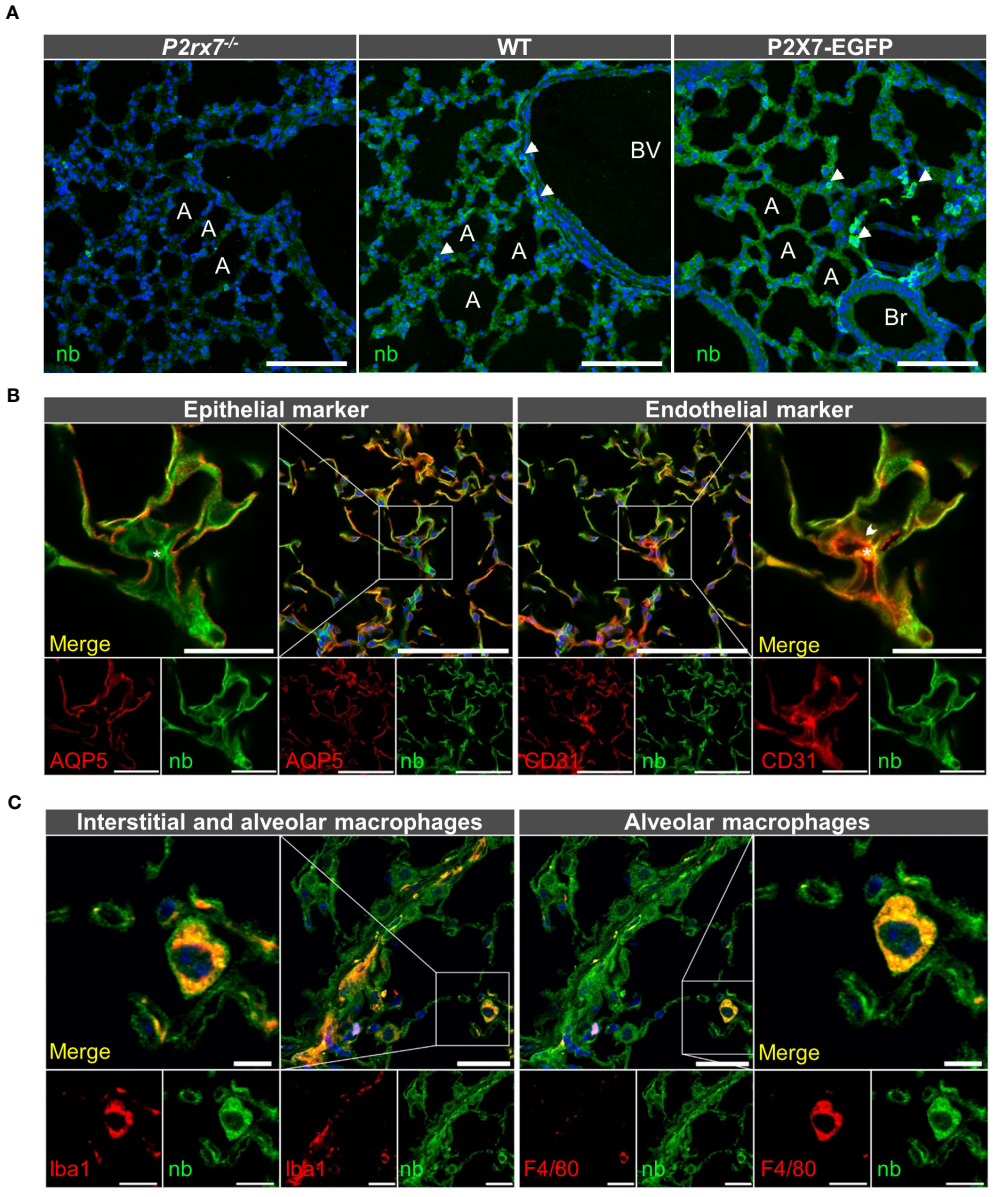
Cell type-specific P2X7 protein expression in the mouse lung. **(A)** 10–20 μm lung cryosections from wt mice, P2X7-EGFP mice, and *P2rx7* ^-/-^ mice as negative control were prepared in parallel and immunostained with a P2X7-specifc nanobody (7E2-rbFc). Arrowheads show brighter cells, most likely macrophages. A = Alveolus, Br = Bronchiole, BV = Blood vessel. Scale bar 100 µm. **(B)** Lung cryosections from wt mice were co-stained with the P2X7 nanobody (7E2 nb-hFc) and antibodies against the epithelial cell marker aquaporin 5 (AQP5) and the endothelial cell marker platelet endothelial cell adhesion molecule PECAM-1 (CD31). Insets show representative areas of differential staining for the two marker proteins. The arrowhead indicates a CD31 positive cell adjacent to a red blood cell (indicated by asterisk). Scale bar 100 µm, inset scale bar 25 µm. **(C)** Co-staining of lung cryosections from wt mice using P2X7 nanobodies (7E2-hFc) and antibodies against ionized calcium-binding adapter molecule 1 (Iba-1) and F4/80 as macrophage markers. Insets show a representative staining of a macrophage positive for both marker proteins. Insets show Iba1 and P2X7-positive cells. Scale bar 25 µm, inset scale bar 10 µm. Nuclear staining with DAPI (in **A, C**) or TO-PRO-3 (in **B**) is shown in blue.

Hence assessing the precise localization of the P2X7 receptor in non-transfected tissues at protein level is crucial, but has been difficult due to a lack of specificity of the available antibodies (66, 67). In particular, P2X4 and P2X7 co-staining in native tissues has been challenging because the commercially available antibodies are derived from the same host. Therefore, to determine sites of possible P2X4/P2X7 interaction in the mouse lung, we generated a P2X7-specific nanobody fused to the Fc domain of human IgG1 (7E2-hIgG hFcAb) and made use of the P2X7-EGFP reporter mouse, which was previously shown to reliably report the P2X7 expression pattern in mouse brain and gut nervous system (54, 68, 69). To confirm that this model also correctly mirrors endogenous *P2rx7* expression in the lung, we first compared immunostainings of lung sections from wild-type (wt), P2X7-EGFP, and *P2rx7* ^-/-^ mice using the previously described P2X7-specific nanobody fused to a rabbit Fc domain (7E2-rbFc, (54)). As seen in **Figure 2A**, both wt and P2X7-EGFP reporter mice present an evenly distributed staining along the lung alveoli, most likely representing epithelial cells, and a stronger signal in single cells, which likely represent macrophages (arrowheads in **Figure 2A**). As expected, sections from wt mice show a lower signal intensity than sections from P2X7-EGFP overexpressing mice. *P2rx7* ^-/-^ mice showed only background fluorescence when imaged under identical conditions. Similar results were obtained using a commercially available P2X7 antibody (**Supplementary Figure 2A**) and with the same nanobody linked to the human Fc-domain (**Supplementary Figure 2B**). To identify the *P2rx7*-expressing cell types, we next performed co-staining with cell type-specific markers. **Figure 2B** shows that the P2X7 staining lining the alveoli overlaps with the epithelial AT1 cell marker aquaporin 5 (70), in support of the previously reported presence of P2X7 in epithelial cells (38, 71). Due to their close association with epithelial cells, presence of P2X7 protein in aerocytes could not be reliably confirmed. However, in regions where a cross-section of a microvessel could be identified, CD31-positive microvascular endothelial cells (arrowhead) show distinct P2X7-positive staining (see insets in **Figure 2B**). In addition, we confirmed that the single cells with more intense P2X7 staining are Iba1-positive macrophages [enlarged inset in **Figure 2C**, (72)]. Only a subset of these are F4/80-immunopositive, most likely representing alveolar macrophages (in **Figure 2C**). Presence of P2X7 in lung fibroblasts was excluded (**Supplementary Figure 3**).

### 3.3 P2X4 and P2X7 show distinct cellular localization

**Figure 3:**
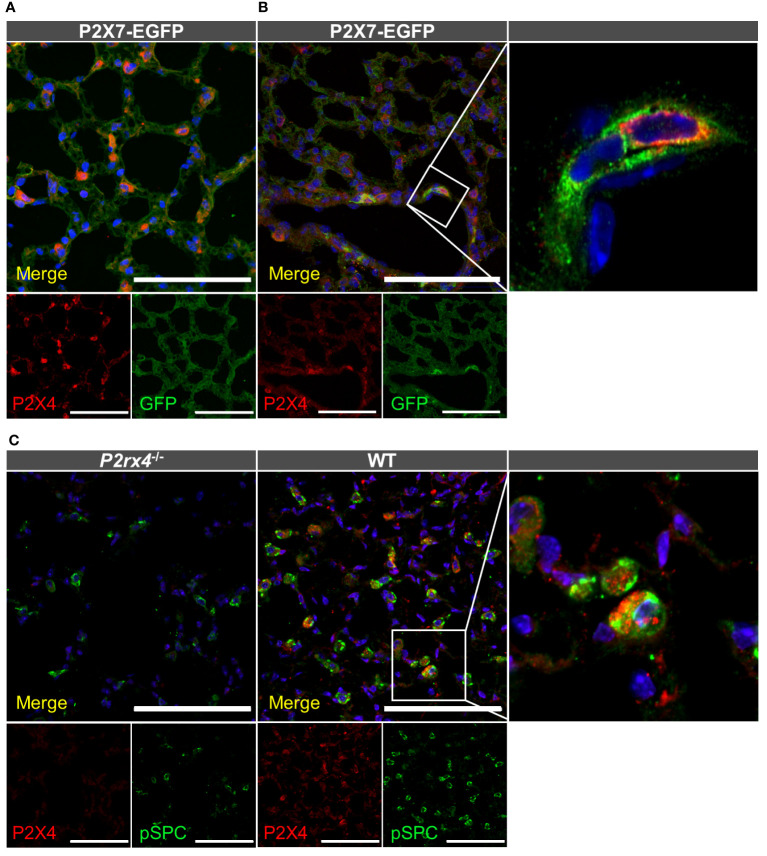
Cellular distribution of P2X4 and P2X7 protein in lung tissue. Cryosections (10–20 µm) of lung tissue from the indicated genotypes were immunostained with anti-GFP (green) and anti-P2X4 (red) antibodies and imaged by confocal microscopy **(A)** and Airyscan **(B)**. Note that the enlarged inset in B was imaged separately **(C)** Specificity of the monoclonal rat anti-mP2X4 antibody in primary cells and co-staining with pro-surfactant protein C as a marker for AT2 epithelial cells. Nuclear staining with DAPI (in **A, B**) or TO-PRO-3 (in **C**) is shown in blue. Scale bar 100 µm. Insets show the representative subcellular distribution of the respective proteins.

After identifying alveolar epithelial cells and macrophages as the dominant P2X7-positive cells in the lung parenchyma, we next performed co-staining of P2X4 and P2X7. To this aim, we first co-stained tissue from P2X7-EGFP transgenic mice with chicken anti-GFP and rabbit anti-P2X4 antibodies (**Figure 3A**). This revealed a clearly distinct cellular distribution of both receptors in the alveolar epithelium. While P2X7-EGFP is consistently localized along the respiratory epithelium, the P2X4 signal is mainly detected in single cells that are larger than AT1 cells and localized at the intersections between alveoli. Only few P2X4-positive cells show clearly overlapping cellular expression with P2X7, and most likely represent macrophages (**Figure 3B**), as both subunits are co-expressed in this cell type [**Supplementary Figure 1**. (13, 73)]. However, a distinct subcellular localization of P2X4 and P2X7 is observed in these cells (**Figure 3B**). Based on the localization and morphology, the majority of P2X4-positive cells most likely represent AT2 cells, in agreement with the modulatory role of P2X4 in secretion of lung surfactant in these cells (33). To confirm the specific localization of P2X4 in AT2 cells, we next used a monoclonal rat anti-P2X4 antibody in combination with an antibody against the AT2 cell-specific marker pro-surfactant protein C (pro-SPC, **Figure 3C**). Despite the evident presence of P2X4 and pro-SPC in AT2 cells, both proteins also show a clearly distinct subcellular localization (**Figure 3C** inset). Whereas the anti-pro-SPC antibody appears to stain larger structures and most of the cell content (in agreement with staining of cytoplasmic pro-SPC), P2X4 shows a more restricted distribution in smaller compartments that most likely represent lamellar bodies. Mature lung surfactant is stored in lamellar bodies of AT2 cells and its exocytotic release has been shown to be facilitated by P2X4-mediated *fusion*-*activated Ca* ^2+^ *entry* (FACE) into lamellar bodies (33). The specificity of the anti-P2X4 antibody was confirmed using lung tissue and macrophages from *P2rx4* ^-/-^ mice as control (**Figure 3C**, **Supplementary Figure S4**).

In conclusion, we detect both P2X4 and P2X7 protein in interstitial macrophages and AT2 cells, in agreement with previous reports (17). However, both appear to have distinct subcellular localization.

### 3.4 P2X4 and P2X7 have distinct subcellular localization

**Figure 4:**
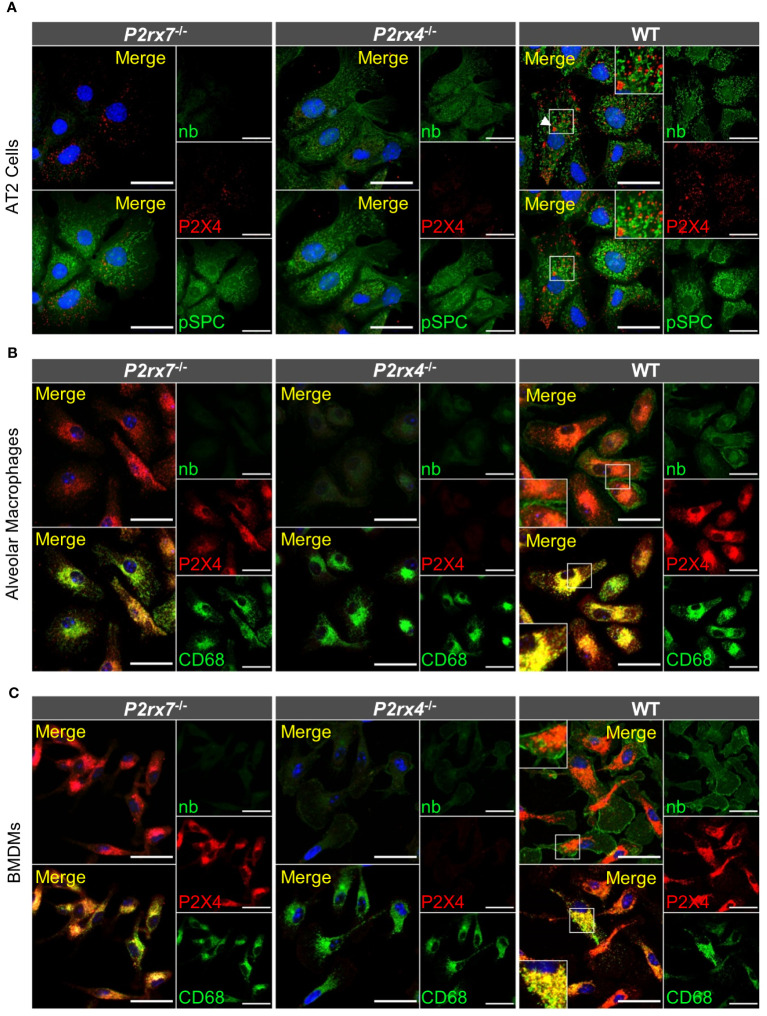
Subcellular localization of P2X4 and P2X7 in primary AT2 cells and macrophages. **(A)** Primary AT2 epithelial cells from wt mice and *P2rx7* ^-/-^, *P2rx4* ^-/-^ controls, were stained 24 hours after plating with the P2X7-specific nanobody (7E2-hFc), rat anti-P2X4 antibody, and anti-prosurfactant protein C (SPC) as an AT2-type cell marker. **(B)** Alveolar macrophages and **(C)** bone marrow-derived macrophages (BMDM) isolated from wt and the respective P2X knock-out control mice were stained with the P2X7-specific nanobody (7E2-hFc), anti-P2X4 antibody, and an antibody against the lysosomal/endosomal marker protein macrosialin (CD68). Nuclear staining with TO-PRO-3 is shown in blue. Scale bar 25 µm. Insets show intracellular puncta and/or membrane structures. Insets in upper panels show areas of intracellular P2X4 staining and comparable areas from control cells. Insets in lower panels show plasma membrane localization. Arrowheads indicate vesicle-like structures, where P2X4 is localized.

**Figure 5:**
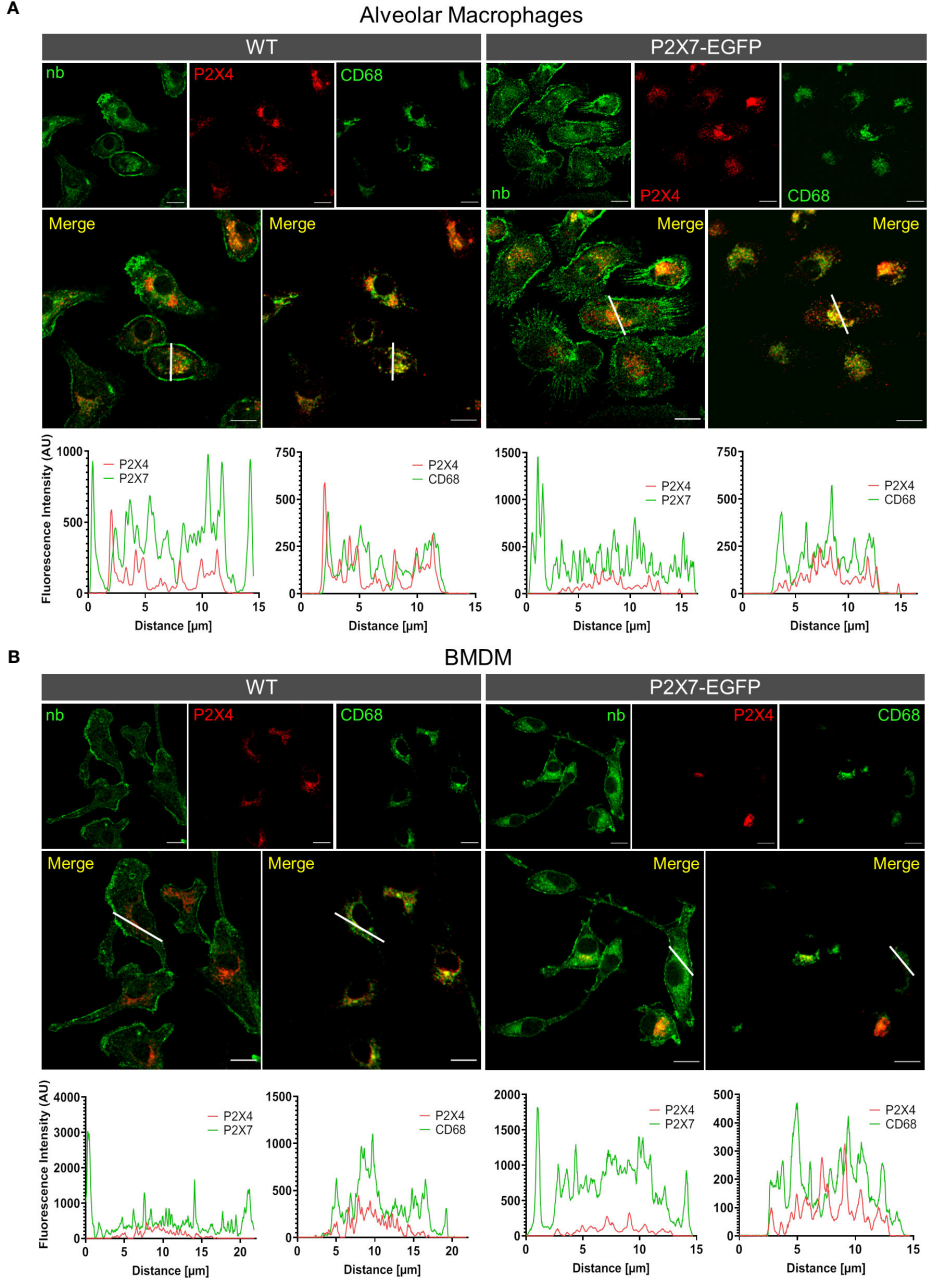
Different subcellular localization of P2X4 and P2X7 in alveolar macrophages **(A)** and bone marrow derived macrophages **(B)** from wt and P2X7-EGFP transgenic mice. Cells were stained with the P2X7-specific nanobody 7E2-hFc, the rat anti-P2X4 antibody, and the monocyte endosome marker CD68. Images were obtained using a Zeiss LSM 880 with Airyscan, and intensity profiles obtained with the built-in image processing software (ZEN black) along the indicated lines. Scale bar 10 μm.

To provide additional support for the cell type-specific localization and the distinct subcellular localizations of P2X7 and P2X4 in AT2 epithelial cells and macrophages and to better estimate possible regions of interaction, we next isolated primary AT2 epithelial cells as well as alveolar and bone marrow-derived macrophages from wt mice and the respective knock-out animals (**Figure 4**). These two types of macrophages were used since interstitial lung macrophages are difficult to obtain and require careful characterization. As expected, P2X4 shows a punctate and clearly intracellular localization in the case of AT2 cells (**Figure 4A**), often lining vesicle-like structures (arrows in **Figure 4A**, **Supplementary Figure S5**). P2X7 staining appears punctate without clear membrane localization (except for cells from transgenic mice (**Supplementary Figure 5**) but does not overlap with P2X4. While the reason for this unexpected intracellular localization is unclear, its presence in AT2 cells was further confirmed by Western blotting (**Supplementary Figure 1B**). In macrophages, a clear membrane localization is seen for both wt and P2X7-EGFP transgenic mice (**Figures 4B, C**, **Supplementary Figure 5**) and, importantly, no co-localization is observed for P2X4 and P2X7 (**Figure 5**). A very similar subcellular distribution of mainly membrane-localized P2X7 and intracellular P2X4 was confirmed in isolated peritoneal and primary microglia, the phagocytic cells of the brain (**Supplementary Figure 6**). Co-staining of alveolar, peritoneal, and bone-marrow derived macrophages with CD68 (**Figure 5**) confirmed the presence of P2X4 in endosomal/lysosomal compartments, in agreement with previous findings, where P2X4 signal overlaps with the signal of LAMP-1 in macrophages, microglia, endothelial cells, and HEK293 cells (14, 19). Taken together, our findings argue against the possibility of substantial physical interactions between both subunits in the plasma membrane of phagocytic cells and might explain the absence of a clear electrophysiological phenotype resulting from their co-assembly (36).

### 3.5 Mutual interrelation between *P2rx4* and *P2rx7* expression?

**Figure 6:**
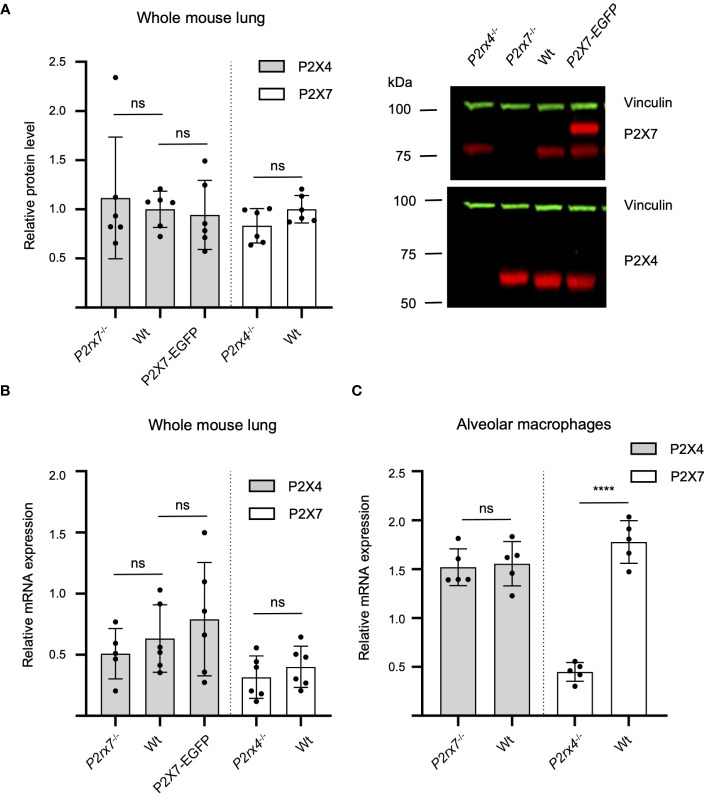
Mutual interrelation of P2X4 and P2X7 expression. **(A)** 50 µg total protein of membrane extracts from lung tissue was separated by SDS-PAGE and immunoblotted with a P2X4- or P2X7-specific antibody (red). Protein levels were quantified via fluorescence intensity of the secondary antibodies and vinculin was used as a loading control for normalization (green). Protein expression of each P2X subtype was not significantly altered by P2X7-EGFP overexpression or genetic ablation of the respective other P2X receptor. Data are presented as mean +/– SD from six animals analyzed in two independent experiments. Significance was analyzed using Student’s t-test. **(B)** Total RNA was isolated from 20–30 mg lung tissue and after reverse transcription quantified in a LightCycler 480 system. RPLP0 and PPIA were used as reference genes to calculate relative P2X4 and P2X7 mRNA levels. **(C)** Total RNA was isolated from alveolar macrophages and PPIA were used as reference genes to calculate relative P2X4 and P2X7 mRNA levels. Significance was analyzed using Student’s t-test. ns, not significant; ****, p<0.0001.

In previous studies, the mutual interaction between *P2rx4* and *P2rx7* expression levels has been analyzed at mRNA and protein levels to identify a possible interrelation. Quantitative reverse transcription (qRT)-PCR data from mouse kidneys showed a significant reduction of *P2rx4* and *P2rx7* mRNA levels in gene-deficient mouse models of the respective other subunit (41). In contrast, a reverse relationship was observed in a study that investigated expression at protein level of both subunits in an alveolar epithelial cell line and found that shRNA-mediated downregulation of one subtype resulted in an increased protein levels of the respective other subtype (38). Therefore, we asked whether overexpression of P2X7 in the BAC transgenic mouse model or the genetic ablation of one of both subtypes influenced protein levels of the respective other subunit in mouse lung tissue. No mutual interrelation on the protein level was observed in the whole mouse lung (**Figure 6A**). Likewise, the mRNA levels of the respective other subunit were not significantly altered by P2X7-EGFP overexpression or genetic ablation of either subunit (**Figure 6B**). Unexpectedly, but in line with the P2X7 staining in primary cells from *P2rx4^-/-^* mice (**Figure 4**) and Western blot analysis from AT2 cells **Supplementary Figure 1B**), a significantly decreased level of *P2rx7* expression was found in alveolar macrophages of *P2rx4^-/-^* mice. (**Figure 6C**). These data were further supported by flow cytometric analyses of alveolar macrophages (characterized as CD11b^+^CD11c^+^CD64^+^CD206^+^), where cell surface-localized P2X7 expression levels were slightly reduced in *P2rx4^-/-^* mice when compared to WT mice (**Supplementary Figure 8**).

## 4 Discussion

Both P2X4 and P2X7 channels have been involved in several lung diseases including pulmonary fibrosis, COPD, and asthma (42, 45–47). Common cellular functions and evidence for and against their interaction have been reported and the formation of P2X4/7 complexes would be of important pharmacological relevance. Since much of this evidence stems from cell culture or overexpression systems, it is still an open question if these interactions take place at physiological expression levels *in situ*. Due to a lack of suitable antibodies, such studies have so far been difficult in native tissues or primary cells. Here, we used P2X7-specific nanobodies in combination with a P2X7-EGFP reporter mouse and P2X4 and P2X7 knock-out controls to investigate for the first time the physical interaction, co-localization, and mutual interrelation of the P2X7 and P2X4 subtypes in the native mouse lung as well as in primary macrophages and epithelial cell cultures. We find no physical association and show that both channels, while co-expressed in macrophages and AT2 epithelial cells, display clearly different subcellular localizations. Finally, we find that deletion of P2X4 reduces *P2rx7* expression in macrophages and AT2 cells while no mutual dependence was observed in whole lung tissue. Our data provide a detailed description of P2X4 and P2X7 protein localization in the mouse distal lung parenchyma and argue against a meaningful physical interaction between both receptors in this tissue.

### 4.1 Do P2X4 and P2X7 receptors physically interact under native conditions?

A possible direct physical interaction and heteromerization of P2X4 and P2X7 receptors is an ongoing debate. The lack of evidence for a physical interaction found in this study is in contrast to many previously described findings where both subunits could be co-immunoprecipitated not only upon overexpression in HEK293 and tsA201 cells but also from a non-transfected cell line derived from alveolar epithelial cells (38) and primary epithelial cells and macrophages (14, 22, 24, 37, 39). Like in non-transfected cells, an almost similar physiological expression and only moderate overexpression levels can be expected in BAC transgenic mice. Along the same line, stably transfected P2X7-EGFP HEK cells presumably express less P2X7 than transiently transfected HEK cells. While we cannot exclude any P2X4/7 complexes below the detection level, the lack of detergent-resistant P2X4/P2X7 complexes is in agreement with a study where native rat tissues were investigated by BN-PAGE (40) and with a very limited overlap of co-localized subunits in native tissues. Thus, the described interaction of P2X4 and P2X7 in *Xenopus laevis* oocytes and HEK cells (24) might be a consequence of P2X4 and/or P2X7 over-expression in recombinant systems and possibly incorrect trafficking or accumulation in intracellular compartments such as the ER and is therefore of minor relevance in native tissue. This is supported by the fact that over-expressed P2X4 subunits show a high tendency to aggregate into oligomers (also seen in **Figure 1**) that likely include P2X7 subunits in case of strong overexpression. Similar findings have been described by Torres et al., who showed that P2X4 constructs were expressed at more than 10 times higher levels than other P2X subtypes in HEK293 cells and this resulted in unspecific interactions (5).

### 4.2 Are P2X4 and P2X7 receptors localized in the same subcellular compartments?

So far, due to limited availability of specific antibodies suitable for co-immunostainings, subcellular localization studies for P2X4 and P2X7 could only be performed by overexpression of tagged proteins and information about possible sites of interaction under native conditions has been lacking.

Compelling evidence for a close physical association of P2X4 and P2X7 receptors comes from co-precipitation studies with primary cells, like bone marrow-derived macrophages. However, despite the fact that both subunits are expressed in the same cell types and seem to be involved in the same signaling pathways, we and others have found a distinctly different subcellular localization. In both recombinant systems and primary cells, the P2X4 subtype was mostly found intracellularly, co-localizing with lysosomal markers (14, 19, 74). In neurons but not primary macrophages, microglia, or vascular endothelial cells heterologously expressed GFP-tagged P2X4 receptors were also detected in the early endosomes (19, 74). P2X7, in contrast, is mainly found at the plasma membrane and only to some extent in intracellular compartments. Intracellular P2X7 signal was found to overlap with an ER marker in transfected NRK cells while the overlap of P2X4 and P2X7 signals in the plasma membrane was only limited (14). Using FRET-analysis and proximity ligation assays, co-localization was detected in transfected HEK cells and *Xenopus laevis* oocytes (24, 35, 39). This co-localization, however, might be limited to overexpressed proteins accumulated in the ER. Moreover, the tendency of some fluorescent protein tags to multimerize needs to be considered.

Nonetheless, trafficking of P2X4 to the plasma membrane is known to occur upon stimulation (e.g., via lipopolysacharide (LPS), (C-C motif chemokine 2 or 12 ionomycin) in microglia and macrophages (37, 75–77) and also in P2X4-transfected NRK (19). Thus, it is possible that in stimulated cells P2X4 receptors localize to the same compartments as P2X7 receptors (e.g. lipid rafts) and this could account for the observed co-precipitation from primary cells. Future studies on primary cells and tissues from our reporter mouse (eg. PLA with stimulated cells) and the presented antibody and nanobody tools might help to clarify this possibility. However, our immunofluorescence data support a pharmacological study in the BV-2 microglia cell line (36) and indicate that under physiological conditions, no, or only very limited amounts of P2X4 receptors are present in the plasma membrane.

### 4.3 Possible functional interactions/interrelations

Our experiments in macrophages show a significant effect of P2X4 deletion on P2X7 expression in alveolar macrophages and AT2 cells (but not *vice versa*), in contrast with previous studies on macrophage-like RAW264.7 cells and bone marrow-derived dendritic cells, which showed that shRNA-mediated downregulation of P2X4 did not affect P2X7 protein levels (21, 23). Together, these studies indicate that depletion of one subunit is neither limiting the expression of the respective other subunit, which would be the case if they are forming obligatory complexes, nor causing a compensatory overexpression of the other subunit, indicative of their interchangeable function in an essential physiological process. However, as mentioned above, data on mutual interrelation of the two P2X subtypes are inconclusive (38, 41) and tissue/cell type-specific differences or effects of cell manipulation might account for these findings and need to be explored. Thus, it has recently been demonstrated that the C57BL/6J *P2rx4* ^-/-^ mice carry a P2X7 SNP (78) that is not present in C57BL/6J wt controls. This passenger mutation has been shown to affect *P2rx7* expression and P2X7 function in T cells (79). Likewise, we cannot exclude that the observed effect on *P2rx7* expression is caused by alterations in the *P2rx7* gene structure as a consequence of *P2rx4* deletion rather than a functional interdependence. In support of this, a recent RNA sequencing study on microglia from P2X4-deficient mice showed that several genes that are located within 7.8 Mbase from the *P2rx4* gene, including *P2rx7*, are down regulated, likely due to chromatine alterations because of the presence of a ß-galactosidase-neomycine cassette (80). Alternatively, P2X4 function in intracellular compartments might be required to regulate P2X7 plasma membrane transport and/or turnover.

Since both P2X4 and P2X7 receptors are ATP-activated Ca^2+^-permeable ion channels and are highly expressed in macrophages, it appears plausible that they can contribute to common signaling pathways and serve similar physiological function, at least if both were present in the plasma membrane. This could well explain the observed mutual influence on current kinetics, pore formation, ROS production and the secretion of mature IL-1beta and IL-18 through the activation of the NLRP3 inflammasome (20, 22, 24, 26). For example, rapid initial P2X4-mediated Ca^2+^ influx was suggested to initiate the P2X7-mediated IL-1b maturation and release (23). Likewise, P2X4 appears to be influence P2X7-mediated autophagy and cell death and both receptors were shown to play a role within the phagosome (19, 25, 26). However, in non-stimulated tissue, we do not see P2X4 in the membrane and the conditions and mechanisms for membrane transport in native tissue need to be further explored. In agreement with our data, (36) found independent activation of both subunits in the BV-2 microglia cell line via whole cell patch clamp. This argues against heteromeric assembly of both receptors, or against a physiological relevance of such.

## 5 Conclusion

The P2X7-EGFP reporter mouse model and novel nanobody tools enabled for the first time the analysis of P2X4 and P2X7 localization and their interaction *in situ* and at physiological expression levels. The presented biochemical and immunofluorescence data argue against a physiological relevance of P2X4/P2X7 complexes in native mouse tissue. We suggest that previously described direct interactions of both subtypes result from over-expression in heterologous systems. Finally, passenger effects due to the close proximity of both genes need to be considered when investigating mutual interdependence.

## Data availability statement

The original contributions presented in the study are included in the article/**Supplementary Material**. Further inquiries can be directed to the corresponding author.

## Ethics statement

The animal experiments were approved by the Regierung von Oberbayern. The study was conducted in accordance with the local legislation and institutional requirements.

## Author contributions

JS-M: Formal analysis, Investigation, Writing – review & editing. LSc: Formal analysis, Investigation, Writing – review & editing. LSa: Formal analysis, Investigation, Writing – review & editing. AR-F: Investigation, Formal analysis, Writing – review & editing. PA: Investigation, Writing – review & editing, Methodology. BR: Investigation, Writing – review & editing, Resources. BZ: Formal analysis, Investigation, Writing – review & editing. JS: Writing – review & editing, Methodology. JH: Writing – review & editing, Investigation. TS: Writing – review & editing, Resources. FK-N: Resources, Writing – review & editing, Funding acquisition. CS-W: Writing – review & editing, Formal analysis. AD: Writing – review & editing, Funding acquisition, Methodology. RK: Conceptualization, Writing – original draft, Investigation. AN: Conceptualization, Funding acquisition, Supervision, Writing – original draft.
