# Supplementary material for: Different localization of P2X4 and P2X7 receptors in native mouse lung - lack of evidence for a direct P2X4-P2X7 receptor interaction
Source: Front Immunol. 2024 Jun 17;15:1425938. doi: 10.3389/fimmu.2024.1425938 (PMC11215518; doi:10.3389/fimmu.2024.1425938)
Supplement: Supplementary file 1 [file DataSheet_1.pdf]

Table S1: Antibodies used for Western blotting and immunohistochemistry

Primary antibodies	Supplier	Cat.# / RRID	Dilution
P2X7<br>(rb pAb)	Synaptic Systems	177003,<br>AB_887755	1:500<br>WB 1:1500
7E2-rbFc / 7E2-hFc	Nolte lab	Nanobody fused to rabbit or human IgG	0.1 µg/mL
P2X4<br>(rb pAb)	Alomone	APR-002,<br>AB_2040058	1:200<br>WB 1:1000
P2X4<br>(RG96 rt mAb)	Nolte lab		0.2 µg/mL
GFP<br>(rb pAb)	Abcam	ab6556,<br>AB_305564	1:2000
GFP<br>(chk pAb)	Thermo Fisher	CA10262,<br>AB_2534023	1:400
GFP<br>(rat 3H9)	Chromotek	3h9-100,<br>AB_10773374	WB 1:1000
Iba1<br>(rb pAb)	WAKO	019–19741,<br>AB_839504	1:100
Iba1<br>(ch pAb)	Synaptic systems	234 009<br>AB_2891282	1:200
Aquaporin-5<br>(rb pAb)	Alomone	AQP-005<br>AB_2039736	1:200
VE-Cadherin<br>(rb pAb)	Cell Signaling	2500<br>AB_10839118	1:200
Podoplanin<br>(gt pAb)	R&D Systems	AF3244<br>AB_2161931	1:200
Prosurfactant protein C<br>(rb pAb)	Merk Millipore	AB3786<br>AB_91588	1:200
F4/80<br>(rt pAb)	Thermo Fisher	14-4801-82<br>AB_467558	1:200
CD68<br>(rt pAb)	Biorad	MCA1957<br>AB_322219	1:200
CD68 (rb pAb)	Abcam	AB125212<br>AB_10975465	1:200
CD16/CD32	BD Pharmingen™	553142<br>AB_394656	1:666
CD45	BD Pharmingen™	553076<br>AB_394606	1:666
CD11b-Bv510<br>clone M 1/70	Biolegend	101245<br>AB_2561390	1:100
CD45-APC-Cy7<br>clone 30-F11	Biolegend	103115<br>AB_312980	1:100
Ly6G-AF700<br>clone 1A8	Biolegend	127621<br>AB_10640452	1:100
CD64-PE-Cy7<br>clone PK136	Biolegend	139313<br>AB_2563903	1:100

CD11c-PE-Dazzle clone RTK2071	Biolegend	117347 AB_2563654	1:100
P2X7-Bv421 clone 1F11	BD Bioscience	744779 AB_2742477	1:100
CD206-FITC clone RTK-2071	Biolegend	141703 AB_10900988	1:100

Secondary antibodies

800CW gt anti-ms	LI-COR	925–32210, AB_2687825	WB 1:15.000
800CW gt anti-rb	LI-COR	926-32211, AB_621843	WB 1:15.000
680RD dk anti-rb	LI-COR	925–68073, AB_2716687	WB 1:15.000
680RD gt anti-rb	LI-COR	925–68071, AB_2721181	WB 1:15.000
680RD gt anti-rat	LI-COR	925-68076, AB_10956590	WB 1:15.000
A594 gt anti-rb	Thermo Fisher	A11037, AB_2534095	1:400
A594 gt anti-ms	Thermo Fisher	A11032, AB_2534091	1:400
A594 gt anti-rat	Thermo Fisher	A11007, AB_10561522	1:400
A546 gt anti-ms	Thermo Fisher	A-11003, AB_2534071	1:400
A488 gt anti-rb	Thermo Fisher	A11008, AB_143165	1:400
A488 gt anti-chk	Thermo Fisher	A11039, AB_142924	1:400
Alexa fluor 405 gt anti-rb	Thermo Fisher	A48254 AB_2890548	1:400
Alexa fluor 488 gt anti-rb F(ab')2	Jackson ImmunoResearch	109-546-088 AB_2337848	1:400
Alexa fluor 647 gt anti-ch	Thermo Fisher	A-21449 AB_2535866	1:400
Alexa fluor 647 gt anti-hs	Jackson ImmunoResearch	127-605-160 AB_2339001	1:400

Table S2: Primers

Name	Sequence	Position
P2rx7 fwd-a	CTGGTTTTCGGCACTGGA	Exon 9
P2rx7 fwd-b	GAGCACGAATTATGGCACCG	Exon 1
P2rx7 rev-a	CCAAAGTAGGACAGGGTGGA	Exon 10
P2rx7 rev-b	GATGCTGTGTCCTAACTTCG	Exon 2
P2rx4 fwd	CCAACACTTCTCAGCTTGGAT	Exon 2
P2rx4 rev	TGGTCATGATGAAGAGGGAGT	Exon 3
PPIA fwd	AGGGTGGTGACTTTACACGC	
PPIA rev	CTTGCCATCCAGCCATTCAG	
RPLP0 fwd	GGACCGCCTGGTTCTCCTAT	
RPLP0 rev	ACGATGTCACTCCAACGAGG	

Fig. S1

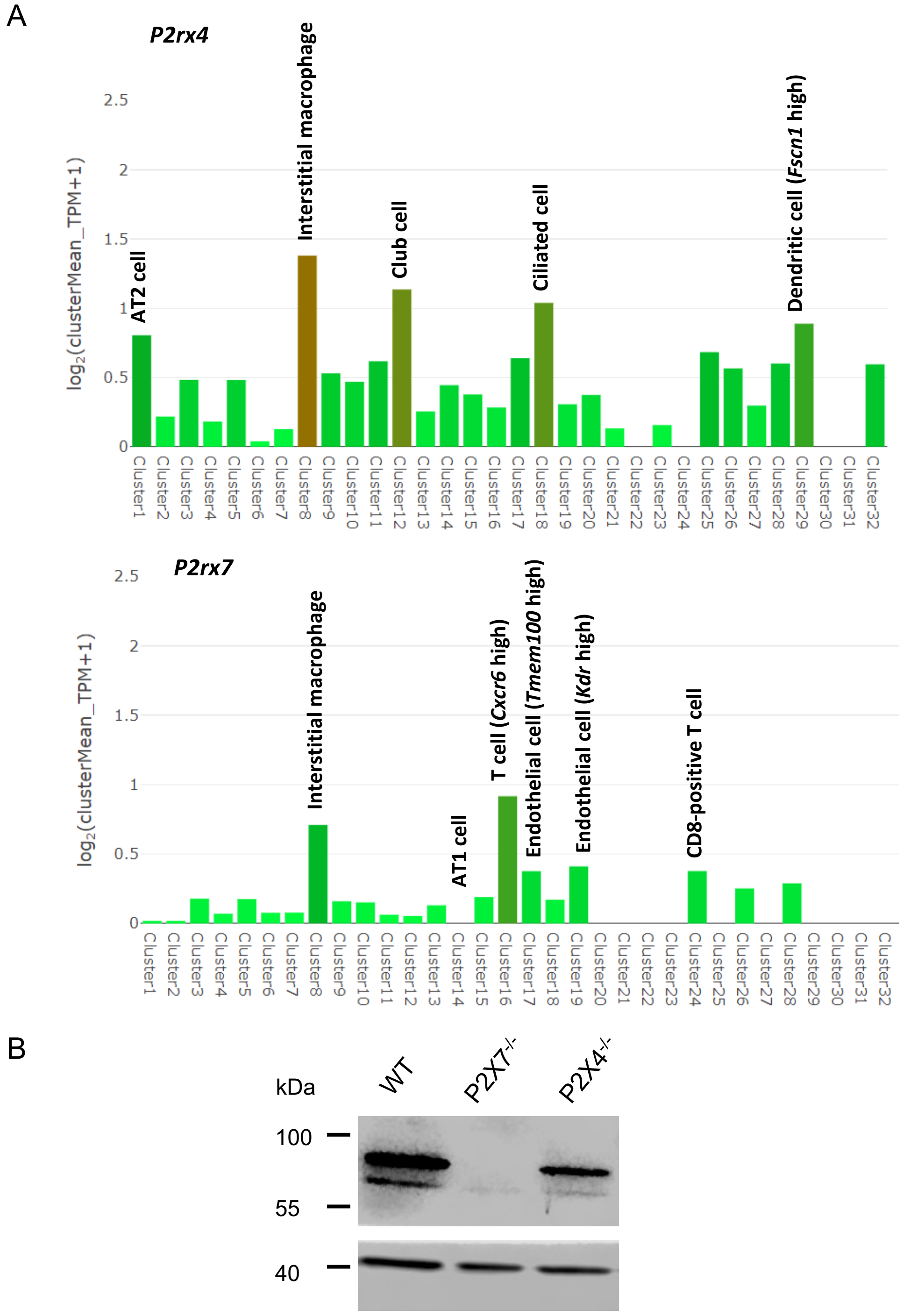

Figure S1: **Expression of P2X4 and P2X7 in mouse lung.**

(A) With the exception of interstitial macrophages, publicly available scRNA-Seq data data indicate comparably little co-expression of *P2rx4* and *P2rx7* in the lung. Data is derived from the Mouse Cell Atlas 3.0 (MCA3.0, Wang R et al, Nucleic Acids Research 51(2), 2023) and was extracted by a search for the two genes within data derived from the tissue “Lung-Adult-lung”. For both genes, the top 5 expressing cell types are specified. For more details, refer to the MCA3.0 webtool (https://bis.zju.edu.cn/MCA/search2.html).

(B) Western blot data confirm presence P2X7 protein in primary AT2 cells despite a limited expression found at the RNA level. 1 x 10^6 AT2 cells from the indicated genotypes were plated on a 6-well plate and after one day, solubilized in RIPA buffer (100 µl/well). 12 µl extract were separated by SDS-Page. P2X7 and β-actin (loading control) were detected with an anti-P2X7 and anti-β-actin antibody (Synaptic Systems, 1:1000, Merck #A3854, 1:10.000 dilution) and anti rabbit peroxicase-coupled secondary antibody (Sigma A6154, 1:10.000 dilution).

Fig. S2

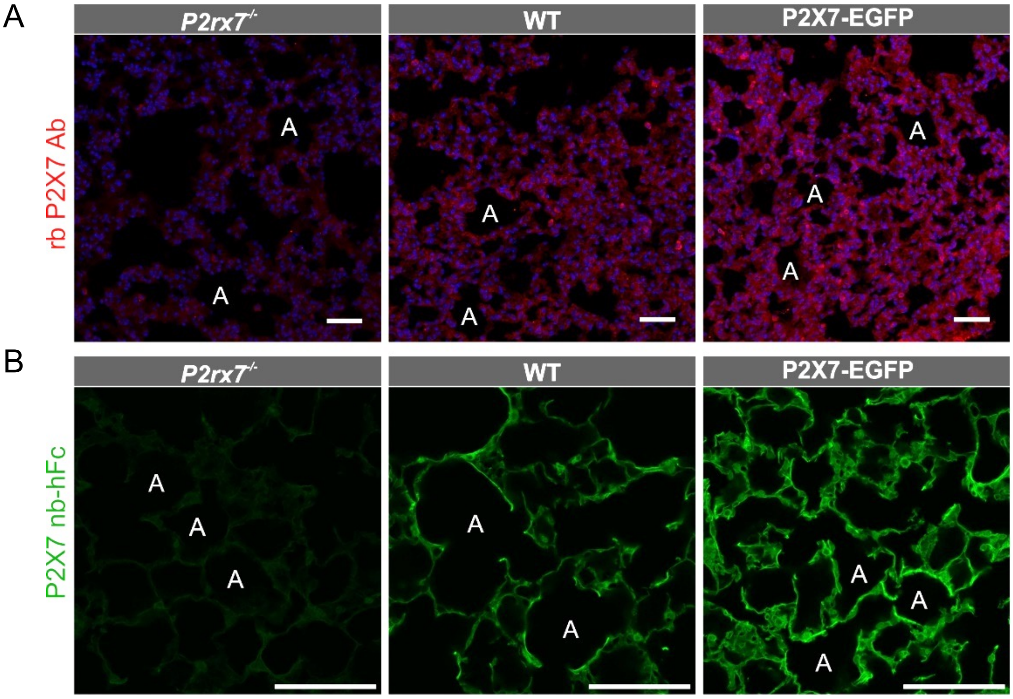

Specificity of P2X7-EGFP expression in lung tissue.

Lung cryosections from the indicated genotypes were stained with (A) an anti-P2X7 antibody (Synaptic Systems 173 003) and (B) the 7E2 P2X7-specific nanobody fused to a human Fc domain (7E2-hFc). DAPI staining is shown in blue. Scale bar 100 µm. A=Alveoli.

Fig. S3

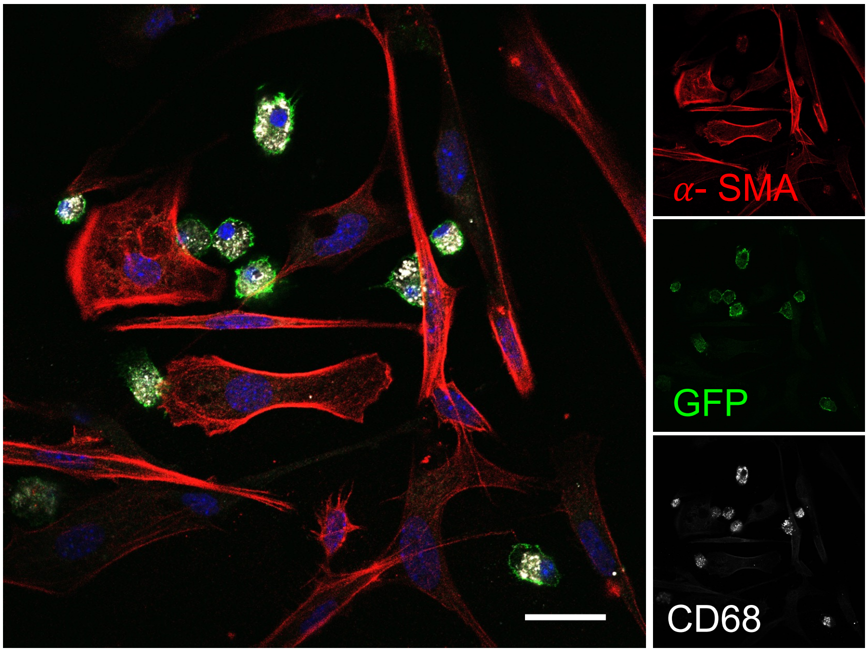

Immunostaining of primary murine lung fibroblasts (pmLF) from transgenic mice with antibodies against GFP and the fibroblast marker *α*-SMA.

Note that cells showing high EGFP expression were identified as contaminating macrophages by staining of the macrophage marker CD68. Nuclear DAPI staining is shown in blue. Scale bar 30 µm. *α*-SMA = alpha- smooth muscle actin.

Fig. S4

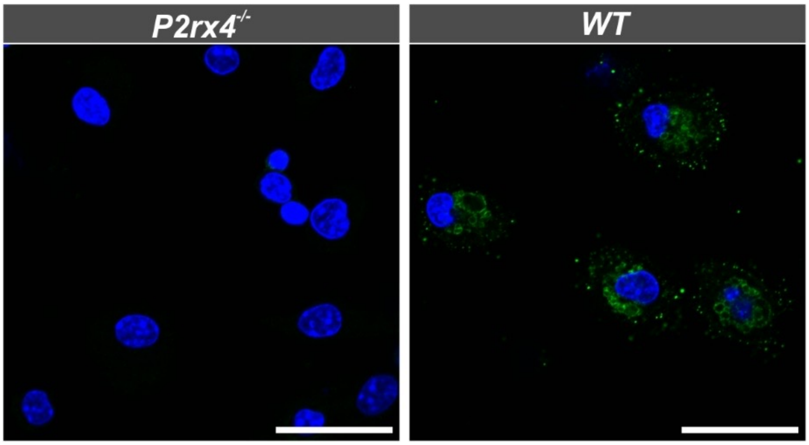

Specificity of the rat anti-mP2X4 antibody.

Macrophages were isolated by alveolar lavage from the indicated genotypes and stained with rat anti-P2X4 (RG96 rt mAb) after 24 hours in culture (37°C, 5% CO2), supplemented with RPMI1640 media containing 10% FCS, Pen/Strep and β-mercaptoethanol. Nuclear DAPI staining is shown in blue. Scale bar 25 μm.

Fig. S5

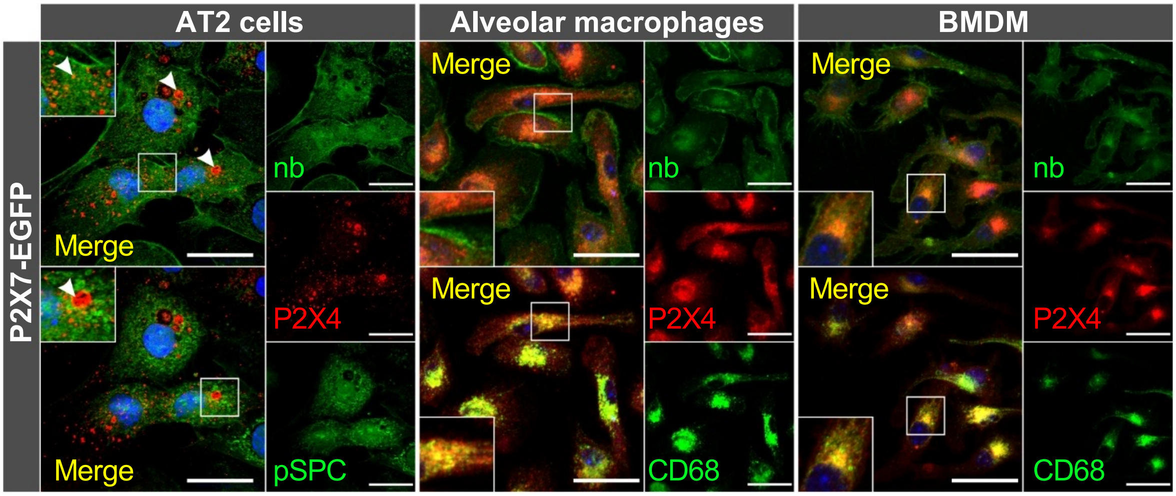

Figure S5. Comparison of the subcellular localization of P2X4 and P2X7 in isolated cells from the P2X7-EGFP mouse. Primary AT2 epithelial cells, alveolar macrophages, and bone marrow-derived macrophages (BMDM) were stained with the P2X7-specific nanobody (7E2-hFc), anti-P2X4 antibody, anti-prosurfactant protein C (SPC) for AT2 cells, and anti-CD68 for the macrophages. Arrowhead show lamellar bodies in AT2 cells, and insets display the distinct membrane pattern for P2X7, and perinuclear puncta staining for P2X4 and CD68. Nuclear staining with TO-PRO-3 is shown in blue. Scale bar 25 µm.

Fig. S6

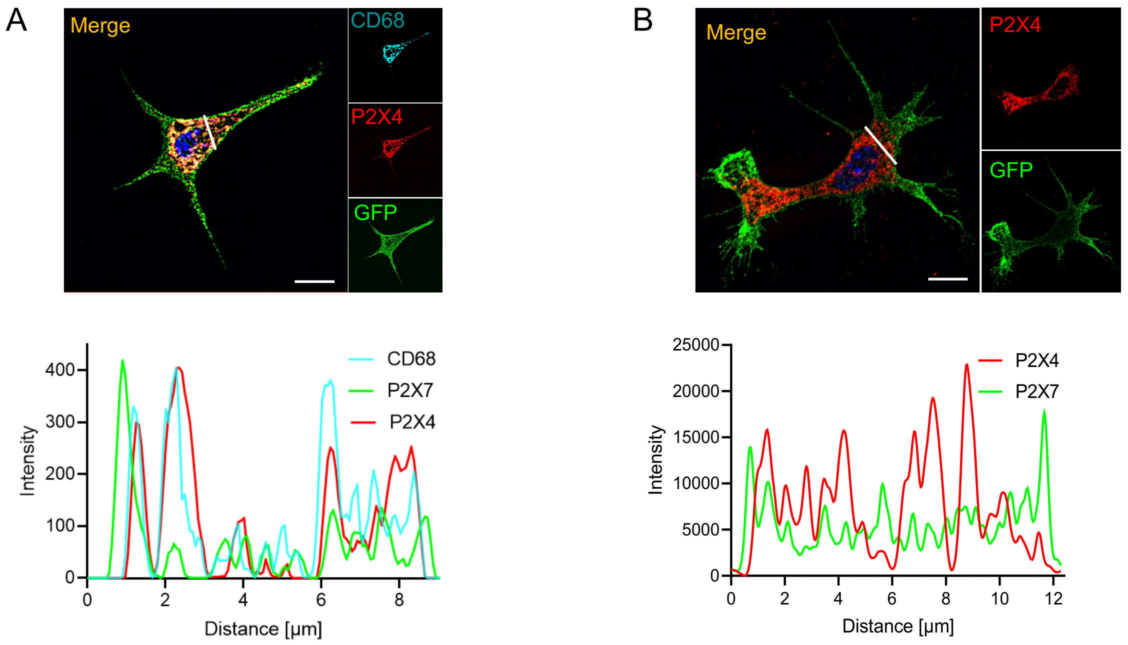

Different subcellular localization of P2X4 and P2X7-EGFP in peritoneal macrophage (A) and primary microglia (B). Cells were stained with antibodies against P2X4 (Alomone, APR-002) and GFP (from chicken, Thermo Fisher, CA10262) and imaged using the Airyscan on confocal laser scanning Zeiss LSM 880. Co-staining with the moncyte endosome marker CD68 was performed in the peritoneal macrophage. Intensity profiles show dominant membrane localization of P2X7 while P2X4 is absent in the membrane. Nuclear DAPI staining is shown in blue. Scale bars 10 µm.

Fig. S7

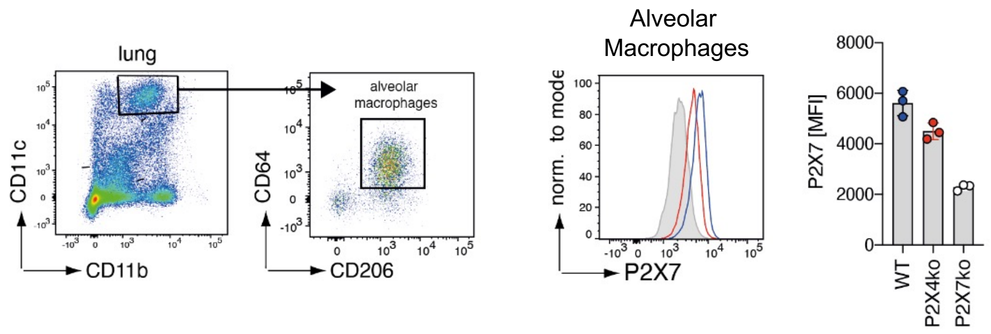

P2X7 on alveolar macrophages (CD11b+CD11c+CD64+CD206+) as determined by by flow cytometry. Mean fluorescence intensity (MFI) of anti-P2X7-Bv421 was calculated for alveolar macrophages from wt (blue), *P2rx4*$^{-/-}$ (red) and *P2rx7*$^{-/-}$ (grey) mice (n = 3).

Mice were anesthesized and sacrificed by CO_2 exposure and cervical dislocation. Lungs were removed, digested for 30 min at 37°C (1 mg/ml collagenase (Roche), 0.1 mg/ml DNAse I (Roche) in DMEM (Gibco)), and passed through a 70 µm cell strainer to generate a single cell suspension. Cells were incubated with ACK erythrocyte lysis buffer on ice and washed once with FACS buffer (PBS + 1% BSA). Percoll (GE Healthcare, 33% with PBS) gradient centrifugation was performed and cells were cell surface stained for 30 min at 4°C with antibodies against CD11b-Bv510, CD45-APC-Cy7, Ly6G-AF700, CD64-PE-Cy7, CD11c-PE-Dazzle, P2X7-Bv421 and CD206-FITC at a concentration of 1:100 (see Table S1). After staining, all cells were washed and resuspended in FACS buffer. Flow cytometric analyses were performed on a BD FACS-Symphony A3 flow cytometer and analyzed by FlowJo™ version 10.8 (BD Life Sciences).

Primary lung fibroblast culture. Mice were euthanized by isoflurane inhalation and transcardially perfused with 5 ml PBS. The lung was extracted, cut into small pieces and transferred into 5 ml DMEM/F-12 (Gibco) supplemented with 1% Pen/Strep (Gibco), 20% FCS, and 1mg/ml collagenase (Biochrom) and incubated for 2h at 37°C and 350 rpm. The obtained cell suspension was filtered through a 70 µm cell strainer and that was washed with 30 ml PBS. Cells were collected by centrifugation (400 x g for 5 min at RT), resuspended in 10 ml DMEM/F-12 with 1% Pen/Strep/20% FCS, and seeded on an uncoated 10 cm petri dish. Cells were cultured at 37°C (95% O2, 5% CO2) and split every 2 days to enrich lung fibroblasts. After 3 passages, cells were plated on coverslips and used for immunofluorescence staining.

Primary microglia culture. P4-P6 mouse pups were decapitated, brains transferred into ice cold DMEM with 1% Penicillin/Streptomycin (DMEM+P/S) and genotypes determined by EGFP fluorescence (Typhoon scanner, GE Healthcare). Five brains were combined in 5 ml of preheated trypsin EDTA solution, cut into small pieces and incubated for 15 min at 37°C in a water bath. Digestion was stopped by addition of 20 ml cell culture medium. Cells were collected by centrifugation (800 x g, 3 min), resuspended in 5 ml DMEM +P/S +10% FCS, triturated with a 1 ml pipet tip, and after addition of 15 ml DMEM +P/S +10% FCS, counted. 7 x 10^6 cells were plated in a T75 flasks and kept at 37°C (95% O2, 5% CO2) with medium changes every 2-3 days. After 14 days, microglia were detached from astrocytes by shaking for 3-4 hrs at 300 rpm and subsequent vigorous tapping. Detached microglia were collected (1500 rpm for 5 min), resuspended in 1 ml DMEM +P/S +10% FCS, plated on poly-L-lysin coated cover slips, and stained the following day.

Isolation of peritoneal macrophages. Mice were euthanized with isoflurane and 5 ml of ice cold HBSS were injected into the peritoneal cavity using a 27 G needle. The peritoneum was gently massaged to detach the cells and the fluid was collected in a syringe and transferred into a 50 ml reaction tube. This step was repeated and the combined cells centrifuged (800 x g for 10 min at 4°C), and resuspended in 2-5 ml DMEM +P/S +10% FCS. 10^5 cells were plated on poly-L-lysin-coated cover slips, cultured overnight at 37°C (95% O2, 5% CO2) and stained.

Image analysis. Cells were stained (see Methods) and imaged using a Zeiss LSM 880 with Airyscan. In Fig. S7, point spread functions for deconvolution of individual channels of confocal micrographs were calculated using the Diffraction PSF 3D (Bob Dougherty, Optinav Inc) plugin applying the respective numerical aperture, wavelength, image pixel spacing and size of the image. Final deconvolution was computed with the plugin Iterative Deconvolve 3D (Optinav Inc).

Flow cytometry. Mice were sacrificed and lungs removed, digested for 30 min at 37°C (1 mg/ml collagenase (Roche), 0.1 mg/ml DNAse I (Roche) in DMEM (Gibco)), and passed through a 70 µm cell strainer to generate a single cell suspension. Cells were incubated with ACK erythrocyte lysis buffer on ice and washed once with FACS buffer (PBS + 1% BSA). Percoll (GE Healthcare, 33% with PBS) gradient centrifugation was performed and cells were cell surface stained for 30 min at 4°C with antibodies against CD11b-Bv510, CD45-APC-Cy7, Ly6G-AF700, CD64-PE-Cy7, CD11c-PE-Dazzle, P2X7-Bv421 and CD206-FITC at a concentration of 1:100 (see Table S1). After staining, all cells were washed and resuspended in FACS buffer. Flow cytometric analyses were performed on a BD FACS-Symphony A3 flow cytometer and analyzed by FlowJoTM version 10.8 (BD Life Sciences).

Schindelin, J.; Arganda-Carreras, I. & Frise, E. et al. (2012), "Fiji: an open-source platform for biological-image analysis", Nature methods 9(7): 676-682, PMID 22743772, doi:10.1038/nmeth.2019.

Dougherty, R. (2005), "Extensions of DAMAS and Benefits and Limitations of Deconvolution in Beamforming", 11th AIAA/CEAS Aeroacoustics Conference (26th AIAA Aeroacoustics Conference)
